# Influence of photoperiod and running wheel access on the entrainment of split circadian rhythms in hamsters

**DOI:** 10.1186/1471-2202-6-41

**Published:** 2005-06-20

**Authors:** Sheila L Rosenthal, Martin M Vakili, Jennifer A Evans, Jeffrey A Elliott, Michael R Gorman

**Affiliations:** 1 Department of Psychology, University of California, San Diego, 9500 Gilman Drive, La Jolla, CA 92093, USA; 2 Department of Psychiatry, University of California, San Diego, 9500 Gilman Drive, La Jolla, CA 92093, USA

## Abstract

**Background:**

In the laboratory, behavioral and physiological states of nocturnal rodents alternate, with a period near 24 h, between those appropriate for the night (e.g., elevated wheel-running activity and high melatonin secretion) and for the day (e.g., rest and low melatonin secretion). Under appropriate 24 h light:dark:light:dark conditions, however, rodents may be readily induced to express bimodal rest/activity cycles that reflect a global temporal reorganization of the central neural pacemaker in the hypothalamus. We examine here how the relative length of the light and dark phases of the environmental cycle influences this rhythm splitting and the necessity of a running wheel for expression of this entrainment condition.

**Results:**

Rhythm splitting was observed in wheel-running and general locomotion of Siberian and Syrian hamsters. The latter also manifest split rhythms in body temperature. Access to a running wheel was necessary neither for the induction nor maintenance of this entrainment pattern. While rhythms were only transiently split in many animals with two 5 h nights, the incidence of splitting was greater with twice daily nights of shorter duration. Removal of running wheels altered the body temperature rhythm but did not eliminate its clear bimodality.

**Conclusion:**

The expression of entrained, split circadian rhythms exhibits no strict dependence on access to a running wheel, but can be facilitated by manipulation of ambient lighting conditions. These circadian entrainment patterns may be of therapeutic value to human shift-workers and others facing chronobiological challenges.

## Background

The critical role of the suprachiasmatic nucleus (SCN) in the orchestration of circadian rhythms represents one of the most successful brain-behavior relationships established in mammalian neurobiology [1]. The cellular and molecular bases of circadian rhythmicity are similarly well characterized [2,3]. Despite these advances, the practical manipulation of human rhythms remains unrealized. Most shift-workers, for instance, fail to reset their circadian pacemakers to effect an alignment of the work shift with the worker's circadian day [4-6]. This misalignment may be a contributing factor in the higher nighttime incidence of industrial accidents and the compromised health of shift-workers [7,8].

In the laboratory under free-running conditions, humans and other mammals typically undergo the rhythmic alternation between physiological and behavioral states appropriate for day (subjective day) and for night (subjective night) [9]. Although individual rhythmic outputs (e.g., locomotor activity, birdsong etc) might be observed to be bimodally expressed, more proximate measures of clock function (e.g., SCN clock gene expression, light sensitivity of the pacemaker, melatonin secretion, etc) tend to alternate unimodally with a period near 24 h [10-12]. One approach to shift-work involves rapid *phase*-*shifting *of the entire circadian program to an optimal compromise between the conflicting demands of work and home schedules [13]. A re-entrainment strategy that manipulates only circadian phase, however, can be easily undone by the entraining effects of bright light exposure in transit to and from work and by exposure to sunlight during the day, both of which serve to reinforce normal diurnal entrainment [6,14]. Additionally, even effective re-entrainment of circadian phase may have costs in terms of the resulting coincidence of subjective night with social demands during the day.

An alternative solution to the problem of shift-work might involve focusing on change in circadian *waveform *instead of phase. Under appropriate lighting conditions, hamsters and mice readily adopt stable, multi-modal entrainment patterns that may meet the requirements of shift-workers for separate intervals of alertness during both the night and the day [15-19]. These entrainment patterns, furthermore, do not require the complete avoidance of daytime exposure to sunlight. Specifically, rodents maintained in 24 h light:dark:light:dark cycles (LDLD) may entrain their circadian pacemakers so that nocturnal locomotor activity is programmed during each of the two daily scotophases 12 h apart (i.e., in anti-phase). A series of behavioral and physiological studies has indicated that this reorganization reflects a global splitting of the circadian pacemaker into two oscillatory components that cycle out of phase [20] (unpublished data). The net result is that, in each 24 h period, the subject goes through two short subjective nights interrupted by two short subjective days. This entrainable split activity pattern occurs in each of three rodent species examined, although the stimulus requirements for maintaining stable entrainment apparently differ by species [18].

To date, two factors have been shown to influence the incidence of rhythm splitting in LDLD cycles. First, animals that engage in robust wheel-running in response to scheduled daily exercise are more likely to split their activity patterns than are animals that remain inactive [16,21,22], and splitting often coincides with a cage change that provokes wheel-running activity [17]. Second, the presence of dim illumination (e.g., 0.004 – 0.10 lux versus complete darkness) during all dark periods markedly increases the proportion of animals that splits in LDLD [17,19]. These two effects may be related as animals are sometimes more active in dim light than in complete darkness, although this cannot wholly account for the facilitative effects of dim light on splitting [23]. In the present experiments, we assessed directly whether the feedback from a running wheel was necessary for the induction and maintenance of rhythm splitting. Additionally, we examined how split rhythms changed as a function of the relative length of day and night. Identification of factors that influence rhythm splitting should serve to clarify underlying mechanisms and inform the design of protocols examining the feasibility of similar entrainment in humans. Finally, body temperature (T_b_) telemetry allowed assessment of circadian markers that may be mediated by mechanisms separate from those underlying locomotor activity [24-27].

## Results

### Experiment #1. Is a wheel necessary for maintenance and induction of splitting in Syrian hamsters?

**Figure 1:**
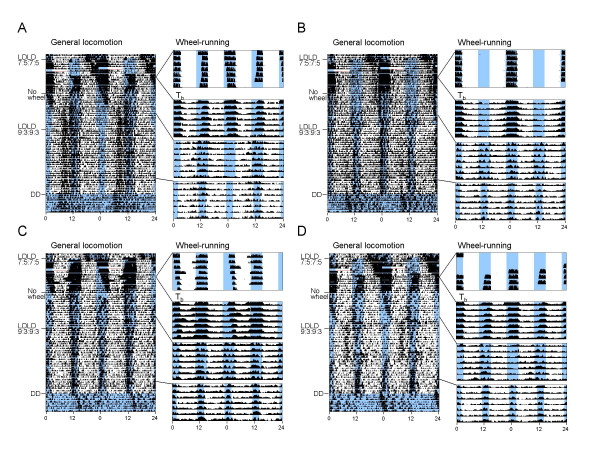
Representative double-plotted general locomotion (GL), wheel-running and body temperature (T_b_) rhythms of Syrian hamsters transferred in Experiment #1 from LD14:10 to LDLD7:5:7:5, to LDLD9:3:9:3 and to constant dim illumination (DD). On the left of each panel is illustrated the GL for the entire experiment. On the right of each panel is shown, respectively, 7 days of wheel-running, T_b _over the same interval, T_b _in LDLD 7:5:7:5 and LDLD9:3:9:3. Lines connect enlarged records on the right with the corresponding GL dates on the left. Times of darkness are shaded blue; GL actograms are scaled as percentile values (ClockLab); wheel running actograms are scaled from 0 to 150 counts/min, and T_b _plots are scaled from 36.0 to 40.0°C. See text for characterization of rhythms of representative animals.

Transfer of Syrian hamsters from standard lighting conditions (14 h light, 10 h dark per day; LD14:10) to LDLD7:5:7:5 and subsequently to LDLD9:3:9:3 yielded the patterns of circadian re-entrainment depicted in Figure 1. In the first two weeks of LDLD7:5:7:5, elevations of general locomotor (GL) activity and T_b _reliably occurred during each of two daily scotophases for several animals (Fig. 1A, C, D). In some cases, these rhythms permanently (Fig. 1A) or transiently (Fig. 1C) fused into a unimodal (i.e., unsplit) pattern following immobilization of the running wheel. Other hamsters adopted bimodal T_b _and GL rhythms in LDLD7:5:7:5 only after immobilization of the running wheel (Fig. 1B). Still other hamsters first exhibited split rhythms under LDLD9:3:9:3. In all cases, split rhythms fused into the unsplit pattern under constant conditions (Fig. 1A–D). Table 1 indicates the number of animals meeting objective criteria for split and unsplit rhythms in each of the experimental intervals. There were no discrepancies between the two measures. Because of limited battery life of the telemeters, the number of reported animals decreased as the experiment progressed.

### Is the T_b _rhythm distinct from that of activity?

**Figure 2:**
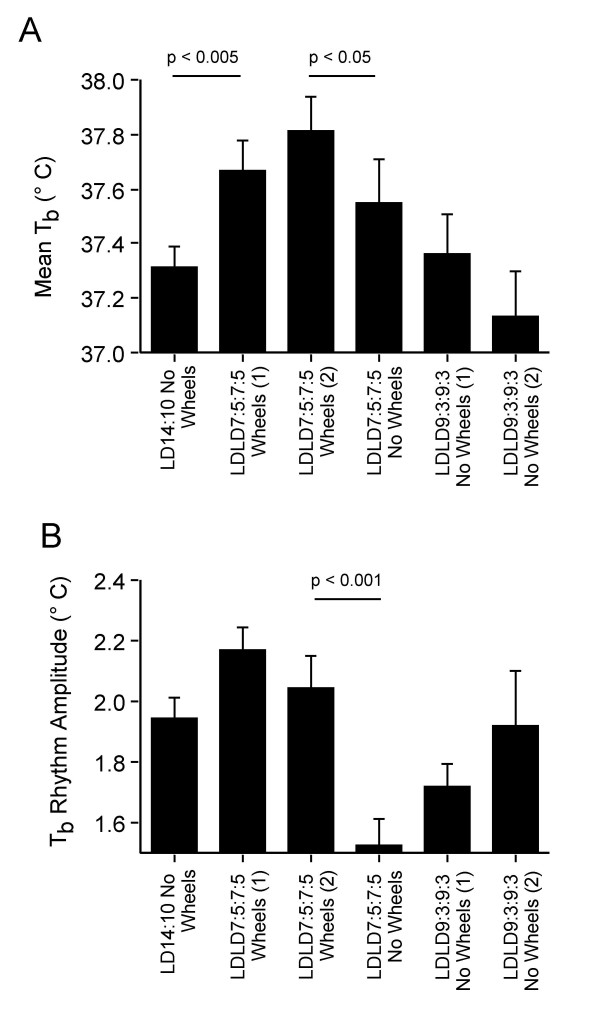
Mean (± SEM) (A) and amplitude (± SEM) (B) of the T_b _rhythm over each of the intervals throughout the experiment. Illustrated are data from 12 hamsters with continuously functioning telemeters, analyzed with repeated measures ANOVA. In some cases (LDLD7:5:7:5 with wheels; LDLD9:3:9:3 without wheels) conditions persisted for several weeks allowing values to be calculated over two intervals separated by cage changes. Means of group pairs below lines with P values differ significantly by paired t-test after correction for multiple comparisons.

Daily mean T_b _and amplitude of the circadian T_b _rhythm varied across the experimental intervals (Fig 2A, B; repeated measures ANOVA, p < 0.001 for both). Mean T_b_, but not T_b _amplitude, increased following initial transfer to running wheels and LDLD7:5:7:5 (p < 0.005; p > 0.05, respectively). Immobilization of wheels 2 weeks later reduced T_b _amplitude (p < 0.001), whereas the reduction in mean T_b _just failed to meet statistical significance after correction for multiple comparisons (p = 0.025). In no interval did mean T_b _or T_b _amplitude differ for split versus unsplit subjects (data not shown).

**Figure 3:**
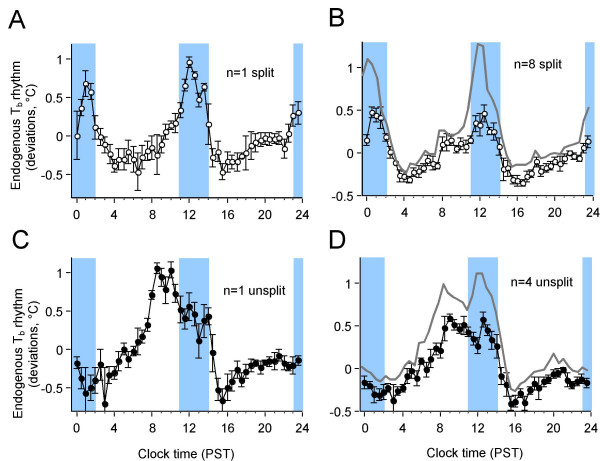
Estimates of the activity-independent T_b _rhythms in individual (A, C) and groups (B, D) of split (A, B) and unsplit (C, D) hamsters maintained in LDLD9:3:9:3. Error bars for individual hamsters represent SEM of 6 values (days) at each time point. Error bars for groups represent between-subjects SEM. Estimates were derived by regressing T_b _against GL and plotting residual values. In (B) and (D) the T_b _rhythm (not removing influence of activity) is plotted (gray lines) in relation to the residual values.

For each individual animal, T_b _was positively correlated with levels of general activity (p < 0.001 in every case; data not shown). Because of likely masking effects of activity on T_b_, we assessed whether there existed an endogenous T_b _rhythm independent of locomotor activity using linear regression to partial out the influence of GL on T_b _rhythms. The residual T_b _not accounted for by GL activity exhibited a reliable daily rhythm in individual-and group-analyzed hamsters during exposure to LDLD9:3:9:3 (Fig. 3). Among split hamsters, there were two unambiguous and equal-sized peaks and troughs daily in the endogenous Tb rhythm (Fig. 3A, B). For unsplit hamsters, there was a substantial rise only during the subjective night and much smaller elevation in antiphase (i.e., during the second scotophase; Fig. 3C, D).

### Experiment #2: Do Siberian hamsters require wheels for splitting?

**Figure 4:**
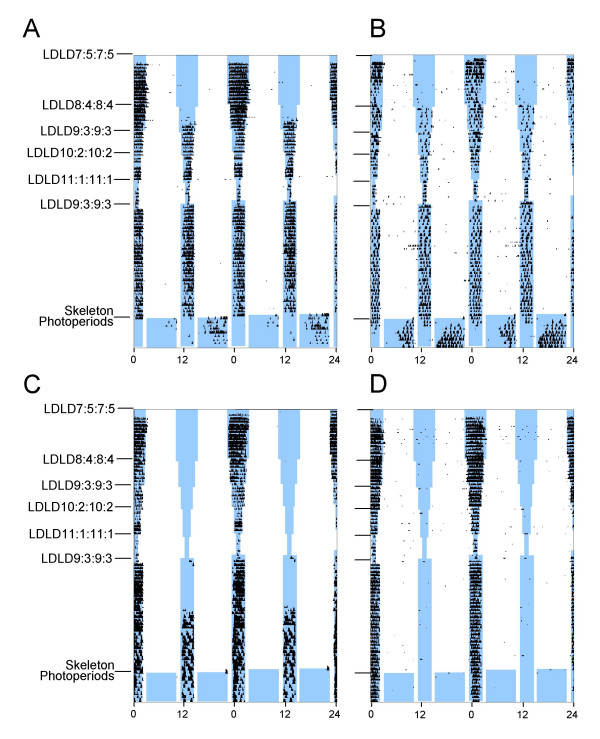
Representative double-plotted wheel-running actograms of Siberian hamsters transferred in Experiment #2 from LD14:10 to LDLD7:5:7:5 and subsequently to other photocycles as noted on the left margins of actograms. Other conventions as in Figure 1. Animals in (A) and (B) demonstrated split behavior very early on in the experiment and remained split until skeleton photoperiods were introduced. The subject in (C) showed no signs of splitting until a second exposure to LDLD 9:3:9:3 and then maintained the split entrainment pattern under skeleton photoperiods. The hamster in (D) exhibited no signs of splitting at any point during the experiment.

**Figure 5:**
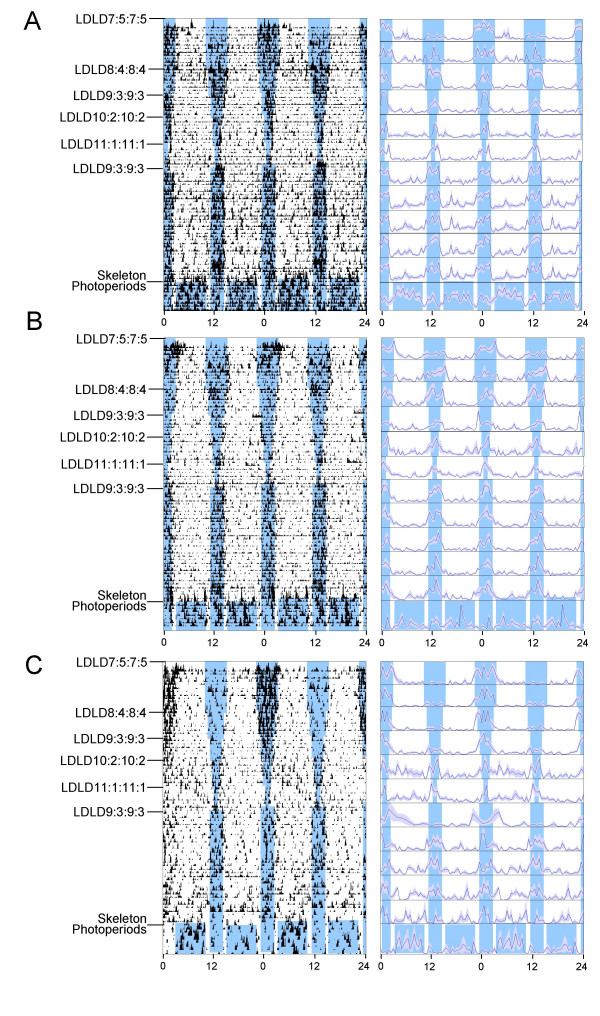
Representative double-plotted actograms (left) and 6-day average activity profiles (± SEM) (right) of hamsters lacking running wheels and monitored by motion detectors. Conventions as in Figure 1. Rhythms of animals in (A) and (B) split early in the experiment but did not remain split under skeleton photoperiods. The hamster in (C) expressed a split rhythm only in LDLD9:3:9:3.

The transfer of Siberian hamsters from LD14:10 to LDLD7:5:7:5 and subsequently to LDLD cycles with progressively shorter scotophases (i.e., LDLD8:4:8:4 etc) induced in individual animals the same split and unsplit rhythms described in Experiment #1 (Figs. 4, 5). A majority of animals with wheels demonstrated split activity bouts for at least one photoperiod condition, but not before LDLD8:4:8:4 (Fig. 4A, B; Table 2). Animals lacking running wheels and instead monitored by passive infrared (PIR) motion detectors did not differ statistically in the incidence of splitting as compared to the wheel group (Fisher's exact test, p > 0.21 in all photoperiods). The number of animals demonstrating split activity tended to increase or remain the same as the length of the scotophases was shortened with the exception of PIR animals in LDLD11:1:11:1. Split rhythms were restored upon return to LDLD9:3:9:3, however.

Of the animals that displayed split rhythms during the second exposure to LDLD 9:3:9:3 (n = 12), 6 maintained a split-activity rhythm when transferred to "skeleton photoperiods" (Fig. 4C). Activity of four of these split animals "phase-jumped" into the two longer (i.e., 7 h) of the four daily scotophases (Fig. 4B, 5C), and one animal that was previously unsplit met the criterion for splitting during the skeleton photoperiod.

**Figure 6:**
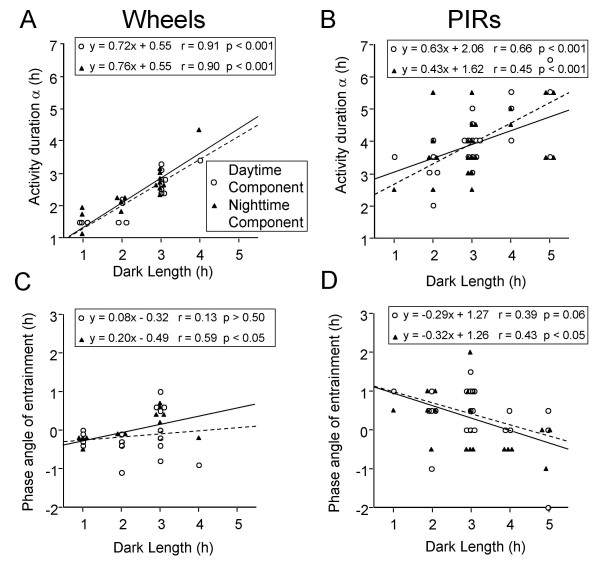
Scatterplots of activity duration (A, B) and phase angle of entrainment (C, D) as a function of entraining photocycle in split hamsters with running wheels (A, C) or without (B, D). Least squares regression lines for the daytime (open circles; dashed lines) and nighttime (filled triangles; solid lines) were calculated separately.

The activity duration (α) of each activity component of split animals varied with the length of the scotophase regardless of whether hamsters had wheels or not (Fig 6, B; p < 0.001 for each). Phase angle of entrainment for the nighttime, but not the daytime component, was positively correlated with scotophase duration for animals with wheels (Fig. 6C). This measure was negatively correlated with scotophase duration in animals lacking wheels (Fig. 6D). For unsplit hamsters, the expected negative correlation between phase angle of entrainment and scotophase duration was obtained only in animals monitored with motion detectors (y = -0.26x + 1.29; r = 0.50, p < 0.005; y = 0.06x – 0.32; r = 0.11, p > 0.45 for PIRs and wheels, respectively).

## Discussion

As reported in previous studies, a substantial fraction of each of two hamster species readily adopts bimodal wheel-running rhythms under 24 h LDLD cycles [18,19,22,23]. Three aspects of the present studies validate the use of measures besides wheel-running to identify these split circadian entrainment patterns. In Experiment #1, the simultaneous recording of T_b_, general locomotor activity and wheel-running yielded essentially identical rhythms within individual animals. Second, separate groups of Siberian hamsters in Experiment #2 monitored by PIR motion detectors and by wheels generated very similar constellations of activity patterns (i.e., split, unsplit, transiently and delayed splits). Finally, the entrainment patterns identified here with motion detectors also mirror wheel-running patterns reported in other studies [17,18]. The persistence of the split entrainment state in some hamsters exposed to skeleton photoperiods is particularly informative as it discounts the possibility that split activity patterns derive from a) a single long subjective night rendered bimodal by negative masking by light or b) a single short subjective night plus a second non-circadian component positively masked by the second dark period. The formal basis of this split pattern has been considered in detail elsewhere [15,17].

The use of measures other than wheel running to identify split rhythms permits testing of the hypothesis that a running wheel is necessary to maintain and/or induce this entrainment pattern. In previous studies, animals that failed to run in a wheel during scheduled daily exposure to novel wheels also failed to later exhibit split home-cage running patterns [21,22]. Additionally, among animals that did not split immediately in LDLD7:5:7:5, we have repeatedly observed abrupt splitting following a cage change during the daytime dark phase [17,23], which typically induces robust wheel-running activity. The present results clearly establish that a wheel is not necessary for splitting in either species: in Experiment #1, split rhythms in LDLD persisted in several Syrian hamsters following withdrawal of wheel access, and other hamsters later adopted split rhythms in the absence of a functional wheel when the length of each of the daily photophases was lengthened. Wheel immobilization was not necessarily responsible for the loss of splitting in some hamsters (see Table 1) because rejoining has been observed in the first weeks of chronic wheel access in similar experiments [17]. In Experiment #2, Siberian hamsters exhibited split rhythms with or without access to a wheel at rates that did not differ between groups. Thus, wheels are necessary neither for maintenance nor for induction of split rhythms. These experiments, however, were not designed with sufficient statistical power to rule out a minor influence of a running wheel on split rhythms.

Whereas it was expected that general locomotion and wheel-running – both measures of activity – would correspond closely, we hypothesized that the T_b _rhythm might differ from activity rhythms if it was controlled by a distinct oscillatory mechanism as suggested in several, but not all, studies [24-27]. As noted above, T_b _rhythms were split in all animals with split activity patterns. Because wheel running likely elevates T_b _[28,29], this bimodal pattern might be expected on the basis of masking alone, and indeed, in this study T_b _rhythms were acutely influenced by access to running wheels. The wheel-running masking account can be dismissed, however, by the persistence of split T_b _patterns after the wheels were immobilized as has been reported with constant light-induced splitting [30]. Highly significant correlations between T_b _and GL values raise the vexing possibility that general locomotor activity, as well as wheel running, can have masking effects [31]. Adjustment of the T_b _data to control for the correlation between T_b _and GL diminished the amplitude but did not eliminate the rhythmicity of the endogenous T_b _rhythm, suggesting that an activity-independent T_b _rhythm is split in the same fashion as that of activity. Of course, the estimate of the activity-independent T_b _rhythm is only as good as the statistical model relating T_b _and GL. Use of a number of different statistical models (e.g., log linear regression; polynomial regression; use of various lag times between T_b _and GL and GL integration times [32]), did not alter our basic conclusion.

As illustrated in Fig. 1 and in previous studies [17], splitting of Syrian hamster rhythms under LDLD7:5:7:5 is not always stable nor was activity always equally distributed between scotophases. In Experiment #1, an increase in splitting incidence was noted after transfer to LDLD9:3:9:3, and no split animal ever rejoined under this photoperiod suggesting that this lighting condition may promote stable splitting. This finding accords with other of our unpublished data from wheel-running hamsters. The same trend was apparent in Siberian hamsters of Experiment #2 where splitting was numerically most common under nights shorter than 5 h. Under the shortest nights, however, split rhythms largely disappeared and were later recovered in LDLD9:3:9:3. These observations are consistent with a model of splitting in which compression of subjective night reduces the stability of the unsplit pacemaker [15,23], and thus a split anti-phase entrainment pattern is preferred. The phase-jumping of split rhythms of several Siberian hamsters in skeleton photoperiods further suggests that the individual split components are not fully stable under 3 h scotophases. Further work will be necessary to determine whether a circadian parameter related to "stability" can be operationalized and convincingly distinguished from known parameters such as period, phase and phase response to light.

The multiple oscillations that underlie splitting under LDLD do not share the same physiological basis as those observed after prolonged exposure to constant light [33]. Whereas the latter case is associated with anti-phase oscillations of the left and right SCN, the former appears to exhibit no such laterality [34](Meyer-Bernstein, unpublished observations). Whether the two LDLD oscillations correspond to the as yet unidentified dual oscillators proposed to underlie photoperiodism, however, remains an open question. At a formal level of analysis, distinct "evening" and "morning" oscillators nicely account for the photoperiodic regulation of activity duration (α) of unsplit animals [12,35]. Because similar photoperiodic regulation of α is seen for each component of the split rhythms in Experiment #2, each of the two split components may be similarly generated by a complex pacemaker comprising multiple oscillators. Supporting this inference, the relationship between phase angle of entrainment and photoperiod for PIR data in Experiment #2 parallels that of unsplit pacemakers [36], albeit non-significantly in the case of the daytime component. Thus, the circadian system likely comprises not just two functional oscillators, but many, which may couple to generate a variety of entrainment states.

Because all mammalian circadian pacemakers likely derive from multiple oscillators in the SCN, we suspect that this split entrainment pattern could be induced in other species under permissive conditions. The practicality and benefit of this entrainment regime to human subjects await evaluation. At least superficially, it may better meet the needs of shift-workers than present phase-shift protocols that attempt to schedule sleep during the real day without achieving full-scale phase shifts of subjective night. With a split circadian rhythm, shift-workers might schedule sleep in two equal intervals sandwiched between a late shift and daytime activity. If the human and hamster pacemakers respond similarly, bright light exposure before and after each sleep phase would reinforce the split pattern rather than compromise entrainment as is the case in some phase-shift protocols. It also remains to be determined whether this circadian arrangement entails any physiological costs, and if so, whether these outweigh potential benefits. From a translational perspective, we are encouraged that split rhythms can be observed under a broader range of conditions than initially identified. Human splitting protocols should consider the influence of factors identified in animal studies: nocturnal illumination, photoperiod and activity levels. Future animal work will assess the influence of age, endocrine status and photoperiodic history.

## Conclusion

Entrainment of novel circadian waveforms is possible with the use of exotic lighting conditions and may represent a productive new strategy for chronotherapeutics. While prior studies point to a role of locomotor activity in inducing split activity patterns, access to running wheels is not necessary to induce or to maintain these entrainment patterns. Instead, split entrainment is influenced by the relative length of light and dark phases of the 24 h LDLD cycle.

## Methods

### General

All procedures were approved by the UCSD Institutional Animal Care and Use Committee. Laboratory bred Syrian hamsters, *Mesocricetus auratus *(of original Harlan stock; HsdHan: AURA, Harlan, Indianapolis, IN), and Siberian hamsters, *Phodopus sungorus *(from a colony maintained at UCSD), were housed at 22 ± 2°C with ad libitum access to water and Purina chow (St. Louis, MO). From birth, Syrian and Siberian hamsters were maintained in 14 h light, 10 h dark daily (i.e., LD14:10; lights on 0300 PST) with photophase illumination of 100 – 150 lux and with no scotophase illumination. Beginning with and continuing throughout the experiments, green light-emitting diodes (LEDs) generated a mean scotophase illumination of 0.027 ± 0.007 lux on the floor of the cage. Photophase illumination remained 100 – 150 lux.

Cage changes, which are potent circadian zeitgebers that facilitate rhythm splitting, always occurred during the first 60 min of the daytime scotophase at intervals of 1–2 weeks. These cage changes defined intervals for statistical analysis, which excluded any data collected 24 h after the perturbation.

### Experiment #1

#### Surgery

At 12 weeks of age, sterile radio-telemeters (Mini-Mitter, Bend, OR) were implanted in the abdominal cavity of Syrian hamsters (16 male/2 female) under Nembutal anesthesia, and hamsters were transferred to individual cylindrical polypropylene cages (20.5 cm diam).

##### Photoperiod and wheel manipulations

At 1000 PST on the tenth day post-surgery, animals were transferred to rectangular polypropylene cages (27 × 20 × 15 cm) equipped with running wheels (17 cm diam) with plastic interleaved through the rungs of these wheels to increase wheel running coordination. Transfer coincided with the onset of the first scotophase of a new LDLD7:5:7:5 cycle (lights off 1000, lights on 1500, lights off 2200, lights on 0300 PST). Animals were divided between two light-tight ventilated chambers with space for 12 cages each.

After 2 weeks in LDLD7:5:7:5, running wheels were permanently immobilized at 0700 PST with plastic zip ties that bound rungs to the top of the cage. After 2 weeks without wheel access in LDLD7:5:7:5, the photoperiod was changed to LDLD9:3:9:3 (lights off 1100, lights on 1400, lights off 2300, lights on 0200 PST) at the beginning of the evening photophase (1500 PST). After four weeks, hamsters were exposed to constant dim illumination (0.027 ± 0.0067 lux) for two additional weeks beginning with the daytime scotophase.

### Experiment #2

At either 21–24 or 41–44 weeks of age, group housed male Siberian hamsters (n = 22) were transferred at 1000 PST to individual cages coinciding with the beginning of a 5 h scotophase of a new LDLD7:5:7:5. Half of the animals, with equal representation of each age cohort, were housed in cylindrical cages equipped with plastic-wrapped running wheels (17 cm diam). The remaining hamsters were housed in rectangular polycarbonate cages (27 × 20 × 15 cm) without a wheel, but equipped instead with a passive infrared motion detector (PIR; Coral Plus, Visonic, Bloomfield, CT) to detect locomotor activity.

After two weeks the LDLD cycle was changed to LDLD8:4:8:4 by reducing the duration of both scotophases by 30 min at each end. At weekly intervals each scotophase cycle was successively shortened by one hour (to LDLD9:3:9:3, LDLD10:2:10:2 and LDLD11:1:11:1). After one week of the latter photocycle, hamsters were maintained for an additional four weeks in LDLD9:3:9:3. Subsequently, each 9 h photophase was replaced with two 1-h "skeleton pulses" to yield LDLDLDLD1:7:1:3:1:7:1:3 for two weeks.

### Data collection

In both experiments, activity and temperature data were collected with Dataquest III hardware (Mini-mitter, Bend, OR) configured for 6 min bins. Prior to implantation, telemeters were calibrated at 36 and 38.5°C. Wheel-running revolutions triggered mechanical sensors that recorded a single count every half rotation. PIR motion detectors registered activity whenever 3 of 27 zones were crossed. Rhythms were plotted and analyzed in ClockLab (Actimetrics, Evanston, IL) with supplementary analyses with Microsoft Excel. Statistical analyses were performed with Statview 5.0 (SAS Institute, Cary, NC).

### Data analysis

#### Incidence of splitting

Different criteria for splitting were required for the different rhythms measured. For wheel running, rhythms were classified as split in any given interval if there were > 15 wheel revolutions for 3 consecutive 6 min bins in both daily scotophases for at least 3 days. As in past studies [18,23], there was never any ambiguity about the split versus unsplit status of wheel-running records.

For PIR rhythms and for telemetered GL and T_b _data, values were smoothed over 30 min bins and the dataset reduced to 48 values per 24 h period (i.e., every 30 min). For classification as split or unsplit during each analysis interval, a 24 h histogram was produced for each hamster by averaging 5–6 days of this reduced dataset. The rhythm was considered split if each histogram scotophase was associated with elevated activity or T_b _levels defined as follows: values exceeding the daily mean by more than one standard deviation for 2 consecutive 30 min bins. In nearly all cases, these objective determinations corresponded with subjective judgments of the rhythms as essentially unimodal (unsplit) or bimodal (split).

#### Analyses specific to Experiment #1

##### T_b _mean and amplitude

In each analysis interval, mean T_b _values were determined by averaging all values over 5–6 days prior to a cage change. T_b _amplitude was calculated as the difference between the maximum and minimum values of 5–6 day histogram calculated from 30 min averages as described above. Repeated measures ANOVA was performed for the 12 animals with uninterrupted telemeter function.

##### Activity-independent component of T_b _rhythm

For each animal with complete data in the second 6-day analysis interval of LDLD9:3:9:3 (n = 8 split, n = 4 unsplit hamsters), 30 min T_b _values were regressed against 30 min GL. Residual values representing the component of T_b _rhythm not accounted for by GL values were retained and averaged at each 30 min time point over 6 days. Alternative models to account for GL yielded very similar results on sample animals. These models included regression of T_b _against log GL values using 30 min values; and use of various lag times and integration intervals for the independent variable [32].

#### Analyses specific to Experiment #2

##### Phase angle of entrainment

In each photoperiod, the phase angle of entrainment for PIR data in Experiment #2 was determined from 5–6 day averages of the 30 min smoothed data. For each component of the split activity rhythm, or for the single component of the unsplit rhythm, activity onset was defined as the earliest point near the L/D transition that exceeded the daily mean. Phase angle was expressed in relation to this transition (positive values indicated activity anticipates lights off).

##### Activity duration

For each scotophase, activity duration (α) was taken as the time difference between the first and last points exceeding the daily activity mean. Both dependent measures (phase angle and activity duration) were regressed against the length of each scotophase for split and unsplit hamsters separately. Because the number and identity of split hamsters changed across the experiment, the dataset was not a proper repeated measures nor were data points fully independent. Recognizing that treating data points as independent would have the effect of reducing statistical power, we opted to do so in linear regression analysis.

## Abbreviations

GL – telemetered general locomotion; LD – 24 h light:dark cycle; LDLD – 24 h light:dark:light:dark cycle; PIR – passive infrared motion detector; SCN – suprachiasmatic nucleus; SEM – standard error of the mean; T_b _– body temperature

## Authors' contributions

SLR and MMV collected and analyzed data for Experiments #1 and 2, respectively and contributed substantially to their design. They contributed equally to this work and are listed alphabetically. JAEv provided significant guidance and assistance to SLR and MMV in data collection and analysis and contributed to both experimental designs. JAEl contributed to experimental design and oversaw the collection of telemetry data. MRG contributed to experimental design, supervised data analysis and worked with SLR and MMV in preparation of the manuscript. All authors edited earlier drafts, and all approved the final manuscript.

## Figures and Tables

**Table 1:** Fraction of Syrian Hamsters Displaying Split Rhythms in Experiment #1†

	LD 14:10 No Wheels	LDLD 7:5:7:5 Wheels (1)	LDLD 7:5:7:5 Wheels (2)	LDLD 7:5:7:5 No Wheels	LDLD 9:3:9:3 No Wheels (1)	LDLD 9:3:9:3 No Wheels (2)
General Locomotion (GL)	0	1/17	13/18	6/18	8/17	10/14
Body Temperature (T_b_)	0	1/17	13/18	6/18	7/16	10/14

†Sample size varies with time due to telemeter malfunction; numbers in parentheses indicate 1st and 2nd intervals of a given photoperiod condition.

**Table 2:** Fraction of Siberian Hamsters Displaying Split Rhythms in Experiment #2

	LDLD 7:5:7:5	LDLD 8:4:8:4	LDLD 9:3:9:3	LDLD 10:2:10:2	LDLD 11:1:11:1	LDLD 9:3:9:3	Skeletons†
Wheels	0/11	1/11	2/11	4/11	4/11	6/11	5/11
Motion Detectors (PIRs)	3/11	3/11	5/11	5/11	1/11	6/11	2/11

†Skeleton photoperiods of LDLD9:3:9:3 (i.e., LDLDLDLD1:7:1:3:1:7:1:3)
